# (1R*,2S*)-2-Nitro-1-(4-nitro­phen­yl)propanol

**DOI:** 10.1107/S1600536812007775

**Published:** 2012-02-29

**Authors:** Xu Zhang, Le Yang, Jun-na Zhang, Wei He

**Affiliations:** a Department of Chemistry, School of Pharmacy, Fourth Military Medical University, Shaanxi Province, Xi’an 710032, People’s Republic of China

## Abstract

The title compound, C_9_H_10_N_2_O_5_, presents a racemic mixture of two enanti­omeric diastereomers. In the crystal, mol­ecules assemble into zigzag chains parallel to the *b* axis [*C*(6) motif] due to the formation of elongated O—H⋯O(N) hydrogen bonds. Of inter­est is the fact that only the aliphatic nitro group is involved in hydrogen bonding and it adopts a *gauche* conformation with respect to the OH group.

## Related literature

For the preparation and synthetic utilities of 2-nitro­ethanols, see: Palomo *et al.* (2005 ▶); Palomo (2007 ▶). For the structure of the closely related 1-(anthracen-9-yl)-2-nitro­ethanol, see: Niazimbetova *et al.* (1998 ▶). For spectroscopic data and chemical properties of the title compound, see: Blay *et al.* (2008 ▶). For hydrogen-bond graph-set notation, see: Etter *et al.* (1990 ▶).
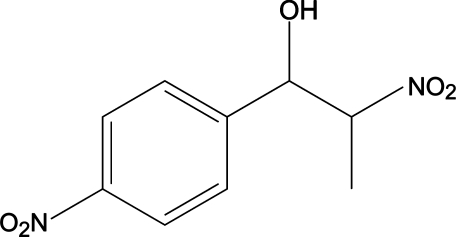


## Experimental

### 

#### Crystal data

C_9_H_10_N_2_O_5_
*M* *_r_* = 226.19
Monoclinic, 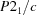

*a* = 7.4013 (15) Å
*b* = 10.504 (2) Å
*c* = 13.681 (3) Å
β = 100.465 (4)°
*V* = 1046.0 (4) Å^3^
*Z* = 4
Mo *K*α radiation
μ = 0.12 mm^−1^
*T* = 296 K
0.40 × 0.28 × 0.14 mm

#### Data collection

Bruker APEXII diffractometer
Absorption correction: multi-scan (*SADABS*; Sheldrick, 1996 ▶) *T*_min_ = 0.954, *T*_max_ = 0.984
5155 measured reflections
1868 independent reflections
1502 reflections with *I* > 2σ(*I*)
*R*_int_ = 0.017

#### Refinement

*R*[*F* ^2^ > 2σ(*F* ^2^)] = 0.045
*wR*(*F* ^2^) = 0.119
*S* = 1.05
1868 reflections
150 parameters
H atoms treated by a mixture of independent and constrained refinement
Δρ_max_ = 0.24 e Å^−3^
Δρ_min_ = −0.16 e Å^−3^

### 

Data collection: *APEX2* (Bruker, 2007 ▶); cell refinement: *SAINT* (Bruker, 2007 ▶); data reduction: *SAINT*; program(s) used to solve structure: *SHELXS97* (Sheldrick, 2008 ▶); program(s) used to refine structure: *SHELXL97* (Sheldrick, 2008 ▶); molecular graphics: *SHELXTL* (Sheldrick, 2008 ▶) and *OLEX2* (Dolomanov *et al.*, 2009 ▶); software used to prepare material for publication: *SHELXTL* and *OLEX2*.

## Supplementary Material

Crystal structure: contains datablock(s) I, global. DOI: 10.1107/S1600536812007775/hg5165sup1.cif

Structure factors: contains datablock(s) I. DOI: 10.1107/S1600536812007775/hg5165Isup2.hkl

Supplementary material file. DOI: 10.1107/S1600536812007775/hg5165Isup3.cdx

Supplementary material file. DOI: 10.1107/S1600536812007775/hg5165Isup4.cml

Additional supplementary materials:  crystallographic information; 3D view; checkCIF report

## Figures and Tables

**Table 1:** Hydrogen-bond geometry (Å, °)

*D*—H⋯*A*	*D*—H	H⋯*A*	*D*⋯*A*	*D*—H⋯*A*
O3—H3⋯O1^i^	0.80 (3)	2.24 (3)	3.010 (2)	162 (3)

Symmetry code: (i) 
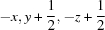
.

**Fig. 1.**
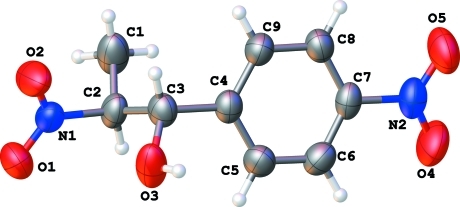
Unsymmetrical unit of I with labeling. Thermal displacement ellipsoids are shown at the 50% probability level.

**Fig. 2.**
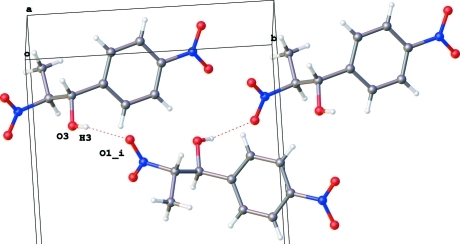
Chain-assembling of I in the crystal lattice (ball-and-stick drawing). Hydrogen bonds are depicted as dashed lines. Only O3, H3, and O1_i atoms are labeled. Symmetry code (i): -x, y + 1/2, -z + 1/2.

## supplementary crystallographic information

### Comment

The title compound, C_9_H_10_N_2_O_5_, I, belongs to the family of β-nitroalcohols which can serve as convenient synthetic precursors for a variety of 1,2-amino alcohols, amino sugars, nitroketones, nitroalkenes, ketones, and other practically important compounds (Palomo *et al.*, 2005; Palomo, 2007).

β-Nitroalcohol I was prepared by a modified procedure described by Blay *et al.*, 2008 (see Experimental). Only one pair of diastereomers is observed among the reaction products, what could be a result of the application of an enantiomerically pure base, natural quinine (see Experimental). In the unsymmetrical unit of I, the N—C(aliphatic) bond is by 0.05 Å longer than the N—C(aromatic) one due to the evident *p*–π conjugation. The aromatic NO_2_ group is slightly twisted in respect to the Ph-ring plane [the C6/C7/N2/O4 dihedral angle equals to 17.9 (3)°]. In crystal lattice, the molecules of I assemble in zigzag chains parallel to the *b*-axis [a *C*(6) motif (Etter *et al.*, 1990)] due to formation of somewhat elongated [2.24 (3) Å] O—H···O(N) hydrogen bonds. Of interest, only the aliphatic nitro-group is involved into the H-binding and adopts a *gauche*-conformation respectively to the OH-group, with the N1/C2/C3/O3 dihedral angle being rather close to 60° [55.2 (2)°]. The same H-binding motif was observed earlier for the case of closely related 1-(antracen-9-yl)-2-nitroethanol (Niazimbetova *et al.*, 1998).

### Experimental

Quinine [(*R*)-(6-methoxyquinolin-4-yl)((2*S*,4*S*,8*R*)-8-vinylquinuclidin-2-yl)methanol, 32.4 mg, 0.1 mmol], Zn(OTf)_2_ [zinc bis(trifluoromethanesulfonate), 36.3 mg, 0.1 mmol], and *p*-nitrobenzaldehyde (151.1 mg, 1 mmol) were dissolved in THF (5 ml). To this solution, excess of nitroethane (10 mmol) was added. After keeping the mixture for 1 h at 253 K, *N*-ethyl-*N*,*N*-diisopropylamine (17.3 µl, 0.1 mmol) was entered and the slurry was allowed to stay for additional 12 h at the same temperature. The resultant solution was concentrated under reduced pressure and then subjected to silica gel flash column chromatography (hexane/ethyl acetate = 10/1), what gave I as a racemic mixture. Purity of the product was proved additionally by the HPLC method. Single crystal of I suitable for the X-ray diffraction analysis was grown from a CH_2_Cl_2_–methanol medium (volume ratio 2: 1). NMR spectral data are in consistence with reported earlier (Blay *et al.*, 2008).

### Refinement

Non-H atoms were refined anisotropically. All H atoms except of the OH group one were treated as riding atoms with distances C—H = 0.96 (CH_3_), 0.98 (CH), 0.93 Å (C_Ar_H), and *U*_iso_(H) = 1.5*U*_eq_(C), 1.2*U*_eq_(C), and 1.2*U*_eq_(C), respectively. The hydroxy-group H-atom was found from the difference Fourier syntheses and refined isotropically.

### Crystal data

**Table:**

C_9_H_10_N_2_O_5_	*F*(000) = 472
*M**_r_* = 226.19	*D*_x_ = 1.436 Mg m^−^^3^
Monoclinic, *P*2_1_/*c*	Mo *K*α radiation, λ = 0.71073 Å
Hall symbol: -P 2ybc	Cell parameters from 1711 reflections
*a* = 7.4013 (15) Å	θ = 2.5–24.3°
*b* = 10.504 (2) Å	µ = 0.12 mm^−^^1^
*c* = 13.681 (3) Å	*T* = 296 K
β = 100.465 (4)°	Prism, colourless
*V* = 1046.0 (4) Å^3^	0.40 × 0.28 × 0.14 mm
*Z* = 4	

### Data collection

**Table:**

Bruker APEXII diffractometer	1868 independent reflections
Radiation source: fine-focus sealed tube	1502 reflections with *I* > 2σ(*I*)
Graphite monochromator	*R*_int_ = 0.017
Detector resolution: 8.333 pixels mm^-1^	θ_max_ = 25.1°, θ_min_ = 2.5°
φ and ω scans	*h* = −8→5
Absorption correction: multi-scan (*SADABS*; Sheldrick, 1996)	*k* = −12→12
*T*_min_ = 0.954, *T*_max_ = 0.984	*l* = −14→16
5155 measured reflections	

### Refinement

**Table:**

Refinement on *F*^2^	Primary atom site location: structure-invariant direct methods
Least-squares matrix: full	Secondary atom site location: difference Fourier map
*R*[*F*^2^ > 2σ(*F*^2^)] = 0.045	Hydrogen site location: inferred from neighbouring sites
*wR*(*F*^2^) = 0.119	H atoms treated by a mixture of independent and constrained refinement
*S* = 1.05	*w* = 1/[σ^2^(*F*_o_^2^) + (0.0497*P*)^2^ + 0.3436*P*] where *P* = (*F*_o_^2^ + 2*F*_c_^2^)/3
1868 reflections	(Δ/σ)_max_ < 0.001
150 parameters	Δρ_max_ = 0.24 e Å^−^^3^
0 restraints	Δρ_min_ = −0.16 e Å^−^^3^

### Special details

**Table:**

Geometry. All e.s.d.'s (except the e.s.d. in the dihedral angle between two l.s. planes) are estimated using the full covariance matrix. The cell e.s.d.'s are taken into account individually in the estimation of e.s.d.'s in distances, angles and torsion angles; correlations between e.s.d.'s in cell parameters are only used when they are defined by crystal symmetry. An approximate (isotropic) treatment of cell e.s.d.'s is used for estimating e.s.d.'s involving l.s. planes.
Refinement. Refinement of *F*^2^ against ALL reflections. The weighted *R*-factor *wR* and goodness of fit *S* are based on *F*^2^, conventional *R*-factors *R* are based on *F*, with *F* set to zero for negative *F*^2^. The threshold expression of *F*^2^ > σ(*F*^2^) is used only for calculating *R*-factors(gt) *etc*. and is not relevant to the choice of reflections for refinement. *R*-factors based on *F*^2^ are statistically about twice as large as those based on *F*, and *R*-factors based on ALL data will be even larger.

### Fractional atomic coordinates and isotropic or equivalent isotropic displacement parameters (Å^2^)

**Table:**

	*x*	*y*	*z*	*U*_iso_*/*U*_eq_	
N1	0.1671 (2)	−0.04487 (15)	0.18274 (12)	0.0528 (4)	
N2	0.3598 (3)	0.67086 (16)	0.05631 (16)	0.0611 (5)	
O1	0.2260 (2)	−0.09283 (15)	0.26305 (12)	0.0718 (5)	
O2	0.0497 (3)	−0.09303 (15)	0.12111 (11)	0.0800 (6)	
O3	0.0003 (3)	0.17439 (15)	0.21466 (14)	0.0757 (6)	
O4	0.4510 (3)	0.72894 (16)	0.12539 (14)	0.0886 (6)	
O5	0.3235 (3)	0.71279 (16)	−0.02787 (15)	0.0872 (6)	
C1	0.3609 (4)	0.0580 (2)	0.07942 (18)	0.0722 (7)	
H1A	0.4478	−0.0088	0.1005	0.108*	
H1B	0.4254	0.1345	0.0684	0.108*	
H1C	0.2816	0.0334	0.0188	0.108*	
C2	0.2477 (3)	0.08152 (17)	0.15862 (16)	0.0522 (5)	
H2	0.3297	0.1121	0.2184	0.063*	
C3	0.0905 (3)	0.17561 (18)	0.13254 (15)	0.0512 (5)	
H3A	0.0060	0.1456	0.0734	0.061*	
C4	0.1629 (3)	0.30631 (17)	0.11196 (14)	0.0464 (5)	
C5	0.2240 (3)	0.3894 (2)	0.18973 (15)	0.0551 (5)	
H5	0.2211	0.3644	0.2546	0.066*	
C6	0.2890 (3)	0.50863 (19)	0.17205 (15)	0.0555 (5)	
H6	0.3295	0.5643	0.2243	0.067*	
C7	0.2926 (3)	0.54335 (17)	0.07561 (15)	0.0489 (5)	
C8	0.2320 (3)	0.46360 (18)	−0.00374 (15)	0.0522 (5)	
H8	0.2347	0.4892	−0.0685	0.063*	
C9	0.1676 (3)	0.34498 (18)	0.01569 (15)	0.0520 (5)	
H9	0.1266	0.2898	−0.0368	0.062*	
H3	−0.076 (4)	0.230 (3)	0.209 (2)	0.103 (11)*	

### Atomic displacement parameters (Å^2^)

**Table:**

	*U*^11^	*U*^22^	*U*^33^	*U*^12^	*U*^13^	*U*^23^
N1	0.0716 (11)	0.0402 (9)	0.0517 (10)	−0.0058 (8)	0.0245 (9)	−0.0030 (8)
N2	0.0612 (11)	0.0396 (10)	0.0843 (14)	−0.0007 (8)	0.0185 (10)	0.0046 (10)
O1	0.0958 (12)	0.0593 (10)	0.0614 (10)	−0.0052 (8)	0.0171 (8)	0.0150 (8)
O2	0.1145 (14)	0.0619 (10)	0.0603 (10)	−0.0379 (10)	0.0069 (9)	−0.0026 (8)
O3	0.0893 (12)	0.0504 (9)	0.1043 (14)	0.0075 (9)	0.0623 (11)	0.0127 (9)
O4	0.1091 (14)	0.0488 (10)	0.1031 (14)	−0.0220 (9)	0.0061 (11)	−0.0073 (9)
O5	0.1046 (14)	0.0596 (10)	0.0953 (13)	−0.0183 (9)	0.0122 (11)	0.0244 (9)
C1	0.0846 (16)	0.0519 (13)	0.0924 (17)	0.0049 (11)	0.0487 (14)	0.0075 (12)
C2	0.0625 (12)	0.0343 (10)	0.0650 (13)	−0.0074 (9)	0.0251 (10)	−0.0013 (9)
C3	0.0542 (11)	0.0440 (11)	0.0587 (12)	−0.0049 (9)	0.0191 (9)	0.0039 (9)
C4	0.0475 (10)	0.0385 (10)	0.0560 (11)	0.0008 (8)	0.0168 (9)	0.0023 (8)
C5	0.0657 (13)	0.0511 (12)	0.0531 (12)	−0.0037 (10)	0.0233 (10)	0.0009 (9)
C6	0.0598 (12)	0.0486 (11)	0.0595 (13)	−0.0036 (9)	0.0151 (10)	−0.0098 (10)
C7	0.0461 (10)	0.0336 (10)	0.0695 (13)	0.0013 (8)	0.0176 (9)	0.0020 (9)
C8	0.0607 (12)	0.0430 (11)	0.0548 (12)	0.0012 (9)	0.0155 (9)	0.0067 (9)
C9	0.0614 (12)	0.0405 (11)	0.0548 (12)	−0.0028 (9)	0.0126 (9)	−0.0021 (9)

### Geometric parameters (Å, º)

**Table:**

N1—O2	1.206 (2)	C2—H2	0.9800
N1—O1	1.215 (2)	C3—C4	1.519 (3)
N1—C2	1.516 (2)	C3—H3A	0.9800
N2—O5	1.217 (2)	C4—C9	1.385 (3)
N2—O4	1.220 (2)	C4—C5	1.387 (3)
N2—C7	1.469 (2)	C5—C6	1.378 (3)
O3—C3	1.407 (2)	C5—H5	0.9300
O3—H3	0.80 (3)	C6—C7	1.374 (3)
C1—C2	1.505 (3)	C6—H6	0.9300
C1—H1A	0.9600	C7—C8	1.380 (3)
C1—H1B	0.9600	C8—C9	1.377 (3)
C1—H1C	0.9600	C8—H8	0.9300
C2—C3	1.519 (3)	C9—H9	0.9300
			
O2—N1—O1	123.54 (17)	O3—C3—H3A	109.4
O2—N1—C2	118.50 (17)	C4—C3—H3A	109.4
O1—N1—C2	117.96 (17)	C2—C3—H3A	109.4
O5—N2—O4	123.24 (19)	C9—C4—C5	118.93 (17)
O5—N2—C7	118.44 (19)	C9—C4—C3	120.81 (17)
O4—N2—C7	118.31 (19)	C5—C4—C3	120.26 (18)
C3—O3—H3	110 (2)	C6—C5—C4	120.86 (19)
C2—C1—H1A	109.5	C6—C5—H5	119.6
C2—C1—H1B	109.5	C4—C5—H5	119.6
H1A—C1—H1B	109.5	C7—C6—C5	118.55 (19)
C2—C1—H1C	109.5	C7—C6—H6	120.7
H1A—C1—H1C	109.5	C5—C6—H6	120.7
H1B—C1—H1C	109.5	C6—C7—C8	122.29 (18)
C1—C2—N1	107.80 (16)	C6—C7—N2	118.74 (18)
C1—C2—C3	116.12 (18)	C8—C7—N2	118.96 (18)
N1—C2—C3	107.79 (16)	C9—C8—C7	118.15 (19)
C1—C2—H2	108.3	C9—C8—H8	120.9
N1—C2—H2	108.3	C7—C8—H8	120.9
C3—C2—H2	108.3	C8—C9—C4	121.22 (19)
O3—C3—C4	113.00 (16)	C8—C9—H9	119.4
O3—C3—C2	105.12 (16)	C4—C9—H9	119.4
C4—C3—C2	110.52 (16)		
			
O2—N1—C2—C1	−69.5 (2)	C3—C4—C5—C6	−179.58 (18)
O1—N1—C2—C1	109.8 (2)	C4—C5—C6—C7	−0.2 (3)
O2—N1—C2—C3	56.6 (2)	C5—C6—C7—C8	0.6 (3)
O1—N1—C2—C3	−124.2 (2)	C5—C6—C7—N2	179.34 (18)
C1—C2—C3—O3	176.23 (17)	O5—N2—C7—C6	−162.9 (2)
N1—C2—C3—O3	55.2 (2)	O4—N2—C7—C6	17.9 (3)
C1—C2—C3—C4	−61.5 (2)	O5—N2—C7—C8	15.9 (3)
N1—C2—C3—C4	177.48 (16)	O4—N2—C7—C8	−163.3 (2)
O3—C3—C4—C9	−146.5 (2)	C6—C7—C8—C9	−0.6 (3)
C2—C3—C4—C9	96.0 (2)	N2—C7—C8—C9	−179.31 (17)
O3—C3—C4—C5	32.9 (3)	C7—C8—C9—C4	0.2 (3)
C2—C3—C4—C5	−84.6 (2)	C5—C4—C9—C8	0.2 (3)
C9—C4—C5—C6	−0.1 (3)	C3—C4—C9—C8	179.61 (18)

### Hydrogen-bond geometry (Å, º)

**Table:**

*D*—H···*A*	*D*—H	H···*A*	*D*···*A*	*D*—H···*A*
O3—H3···O1^i^	0.80 (3)	2.24 (3)	3.010 (2)	162 (3)

Symmetry code: (i) −*x*, *y*+1/2, −*z*+1/2.
